# Regarding: ‘prevalence, characteristics and measurement of somatic symptoms related to mental health in medical students: a scoping review’

**DOI:** 10.1080/07853890.2025.2554925

**Published:** 2025-09-03

**Authors:** Rajah Darwish, Marina Baher Zakhary, Kaleigh Monét Wingate

**Affiliations:** Florida State University College of Medicine

We read with great interest the article by Sperling et al. [1] published in Annals of Medicine, which synthesizes the prevalence and characterization of somatic symptoms among medical students and their close association with psychological distress [1]. As current fourth-year medical students, we commend the authors for highlighting a frequently underrecognized dimension of trainee well-being.

The review notes that somatic symptoms, especially musculoskeletal pain, headaches, gastrointestinal disturbance, and fatigue, are common in medical students and closely associated with psychological stress [1]. This resonates deeply with our lived experience. One symptom that merits specific attention in this context is globus pharyngeus. During a period of intensive examination stress, one of us developed a persistent, non‑painful sensation of a ‘lump’ in the throat that fluctuated with stress levels. The functional impact was substantial: frequent throat‑clearing, difficulty sustaining oral presentations, reliance on sips of water, and repeated bathroom breaks that disrupted workflow and undermined performance.

Seeking answers, she pursued extensive medical evaluation, including swallow studies and specialist consultations, costing her hundreds in out-of-pocket charges. Her workup was negative, reinforcing that the etiology of the symptom was likely stress-related somatization rather than primary pathology [2]. Although benign in pathophysiology, the practical and emotional toll was not insignificant. This experience mirrors the review’s observation that somatic symptoms can meaningfully impair function even in the absence of structural disease [1].

From a methodological standpoint, we invite the authors to comment on two practical issues that, if addressed, could strengthen future work in this area. First, commonly used somatic symptom inventories vary in content and may not systematically capture throat‑focused phenomena such as globus; clarification on whether instruments used across included studies explicitly assessed this domain would be helpful. Second, the heterogeneity of definitions and measures across studies complicates pooled prevalence estimates; we would welcome the authors’ perspective on whether a core, trainee‑relevant symptom set (e.g. a standardized subset from widely used scales) could facilitate cross‑study comparability.

Finally, we suggest a curricular application of the authors’ findings. Brief psychoeducation on somatization; clear, accessible guidance on when to seek medical evaluation; and provision of low‑cost, high‑yield wellness resources (scheduled breaks, physical activity, mindfulness options, and confidential counseling) could de‑stigmatize these presentations, reduce unnecessary diagnostic pursuits, and encourage timely support‑seeking. Given the potential for educational and clinical disruption, stress‑related somatic symptoms deserve explicit inclusion in orientation and clerkship transition programming.

## Data Availability

No new data were generated or analyzed in support of this correspondence. Data sharing is not applicable to this article.
